# Supplementary material for: Metabolomic analysis of murine tissues infected with Brucella melitensis
Source: PLoS One. 2025 Jan 27;20(1):e0314672. doi: 10.1371/journal.pone.0314672 (PMC11771894; doi:10.1371/journal.pone.0314672)
Supplement: S1 Table — (DOCX) [file pone.0314672.s001.docx]

**Table S1. Primers used in this study**

| **Primer name** | **5’-3’ sequence** | **Purpose** |
| --- | --- | --- |
| bmei0265-up-F* | GCGCCAGAAAGCTTCCTGCAGGATATCGTGAAAGTCATGCGAAAAACGCAGG | Cloning fragment upstream of *bmei0265* |
| bmei0265-up-R | ATCGGCCATCTACTTGCCTGCG | Cloning fragment upstream of *bmei0265* |
| bmei0265-cat-F^ | CGCAGGCAAGTAGATGGCCGATGTGTAGGCTGGAGCTGCTTC | Cloning chloramphenicol resistance gene |
| bmei0265-down-F^#^ | GGAATAGGAACTAAGGAGGATATTCATATG CAGAAGTAAATTGCCTGATATATTAGC | Cloning fragment downstream of *bmei0265* |
| bmei0265-down-R* | CCAAGCTACGTAATACGACTCACTAGTGGG CCTTCTGGTTACGGCGCTGGATGG | Cloning fragment downstream of *bmei0265* |
| bmei0265 seq F | AATGGTGAGCAGATCACCCTCG | Screening of *bmei0265* mutant |
| bmei0265 seq R | CAGCAAAAATGCGAAGCGG | Screening of *bmei0265* mutant |

*Underlined regions denote homology to pNTPS139

^Underlined region denote homology to *bmei0265* upstream fragment

^#^Underlined region denote homology to cloned chloramphenicol resistance gene

All primers were from IDT, Coralville, IA
